# Unboxing Electronic Flashcards: An Introduction to the Design Elements of Anki for Medical Education (DEAME) Framework

**DOI:** 10.1007/s40670-025-02518-1

**Published:** 2025-11-28

**Authors:** Emily A. Balczewski, Philip D. Barrison, Asavari Rajpurkar, Kirsten Fiestan, Pratik S. Vadlamudi, Matthew Sparling, Zach Landis-Lewis, Alexandra H. Vinson

**Affiliations:** 1 University of Michigan Medical School, University of Michigan, Ann Arbor, MI USA; 2 Department of Learning Health Sciences, University of Michigan Medical School, Ann Arbor, MI USA; 3 Department of Obstetrics and Gynecology, University of Utah School of Medicine, Salt Lake City, UT USA; 4 Department of Internal Medicine, University of Iowa Carver College of Medicine, Iowa City, IA USA; 5 Department of Anesthesiology, Northwestern University McGaw Medical Center, Chicago, IL USA; 6 Department of Anesthesiology, Beth Israel Deaconess Medical Center, Boston, MA USA; 7 Department of Computational Medicine and Bioinformatics, University of Michigan Medical School, Ann Arbor, MI USA

**Keywords:** Anki, Medical education, Electronic flashcard, New Taxonomy of Educational Objectives, Knowledge representation

## Abstract

Electronic flashcards (EFs) have rapidly ascended in popularity as an active learning resource among medical learners. Despite their significant popularity, no unifying terminologies exist to support clear practices and communication about the design of EFs between learners, educators, and researchers. This study proposes a framework extending *The New Taxonomy of Educational Objectives* (*NT*) to offer a robust terminology for describing EF design. Thirteen hundred EFs from six popular undergraduate medical education decks were sampled and qualitatively analyzed using a multi-phase design and flexible approach to thematic analysis. Through an iterative process of induction, deduction, and reconciliation, general design dimensions (“categories”) and specific design elements (“codes”) of EFs were identified. These categories and codes were organized into a unifying framework with accompanying definitions, examples, and possible implications for student-centered design. The Design Elements of Anki for Medical Education (DEAME) Framework contains seven categories and 48 codes that provide a robust foundation for conceptualizing EF design. Two categories describe the multimedia and metadata elements in EFs. Three categories describe how EFs formulate questions and answers. Two categories extend the *NT* to describe the types of knowledge and ways of thinking EFs can elicit. The DEAME Framework provides a much-needed terminology to describe the significant design variety in EFs for medical education. This terminology enables future educators, researchers, and learners to better develop and communicate best practices for creating high-quality EFs that align with medical curricula.

Electronic flashcards (EFs) have become a widely popular study tool among medical learners in recent years, with some studies showing adoption rates in undergraduate medical education (UME) cohorts as high as 85%, rivaling the use of traditional curricular resources like lectures [1, 2]. As EFs continue gaining traction in medical education, all educational stakeholders—including learners, educators, and researchers—will benefit from a greater understanding of how EFs are designed to facilitate student learning.

## Introduction

The traditional understanding of paper flashcards, the predecessor to EFs, suggests that their benefits as a study tool are twofold: (1) integrating knowledge through the process of flashcard creation and (2) rehearsing factual knowledge through the process of flashcard revision [3]. However, recent scholarship suggests that learners might engage with EFs differently than this traditional understanding. For one, several studies in medical education hint that EFs can elicit higher-order learning processes beyond simple recall and often contain procedural instead of only factual knowledge [4–9]. Furthermore, studies suggest that medical learners minimally create their own EFs and instead use EF decks created by other learners that are distributed widely on social media platforms like Reddit and AnkiHub [10–12].

To effectively use EFs as a learning tool in medical education, all stakeholders—including learners, educators, and researchers—need a shared understanding of EFs and how they facilitate knowledge acquisition through their design. However, no consensus definition of EFs exists, which is further complicated by the wide variety of EF design elements, including heterogeneous question types, question elements, explanatory information, and metadata [5, 7, 8, 13–23].

To wrangle the complexities of EF design into a single and robust terminology for use by learners, educators, and researchers, we created the Design Elements of Anki for Medical Education (DEAME) Framework using a flexible, inductive-deductive approach to thematic analysis. DEAME organizes and describes the design elements (e.g., types of questions, answers, multimedia, and metadata) found in a sample of EFs used by medical trainees. Additionally, incorporated into DEAME is a novel extension of *The New Taxonomy of Educational Objectives* (*NT*)—an update to *Bloom’s Taxonomy*—that defines the types of knowledge in EFs and how EFs test that knowledge [24]. The DEAME Framework can serve as a starting point for learners and educators, regardless of prior experience with EFs, to understand the current state of EFs in medical education and work towards their improvement as a learning tool.

## Background

### What Are Electronic Flashcards?

Electronic flashcards—also called e-flashcards, digital flashcards, virtual flashcards, and interactive flashcards—facilitate active engagement in learning. EFs typically have a question (“front”) and answer (“back”) component like traditional paper flashcards. The electronic nature of EFs also allows for additional capabilities, such as multimedia, analytics, gamification, and spaced repetition. These electronic features are facilitated by EF software platforms, such as the free and open-source Anki application [25]. Additionally, many online communities, such as Reddit’s /r/medschoolanki/ [26] and the paid service AnkiHub [27], provide spaces to share EF decks and circulate advice about EF use.

Research has yielded preliminary insights into learner behaviors, attitudes, and outcomes around EFs for the health professions, including medical education [28]. Learners usually demonstrate high uptake (> 50% of the study population) of EFs when offered [4, 10, 13, 14, 16–18, 22, 29–33]. However, they may use EFs in differing ways, such as for massed [14, 17, 20, 31] vs. spaced/distributed practice [8, 16, 34, 35] and learning new knowledge [4, 29, 36] vs. rehearsing old knowledge [8, 34, 37]. The attitudes towards EFs among learners are largely positive, with a handful of studies suggesting that EFs are perceived as helpful [4, 7, 10, 16, 22, 34, 36–40], easy-to-use [4, 7, 10, 19, 36, 39], and confidence-boosting [10, 33, 36, 41]. When prospectively assessing the impact of EF use on academic outcomes, most studies [5, 14, 16, 17, 23, 32, 37, 38, 42]—including all three with a randomized, controlled design [19, 41, 43]—show improvements in exam scores or clinical performance among EF learners. A dose-dependent effect of EF use is suggested by recent studies, which generally found positive and significant correlations between academic outcomes and specific usage metrics, such as time spent using EFs or the number of new cards reviewed [14, 17, 37, 39, 42, 44–46].

Notably, the interpretation, extensibility, and replicability of the growing EF literature remain challenging because many studies lack a consistent terminology to report the details of design, content, and scope of EFs used by learners [28].

### What Is *The New Taxonomy of Educational Objectives*?

*Bloom’s Taxonomy* (*BT*) is often the de facto choice to characterize the types of thinking elicited by learning objectives and assessments [47]. However, a known limitation of *BT* is that its six levels contain a mix of types of knowledge and ways of thinking with that knowledge [48]. To address this limitation and others, several updates to *Bloom’s* have been proposed, including *The New Taxonomy of Educational Objectives* (*NT*), which contains two domains that separately describe knowledge types and cognitive processes [24]. EFs themselves may elicit different types of knowledge [9] and thinking skills [4–8, 40] and, therefore, benefit from a description by the *NT*’s two-dimensional structure. Marzano and Kendall suggest that intentionally designed learning resources should be closely related to learning objectives [24, 49]. Within medical education contexts, a closer consideration of how EFs could be designed to align with course content would be assisted by a framework that clearly delineates the design features of EFs, including how EFs represent forms of medical knowledge and thinking skills. In this manuscript, we build on the work of Marzano and Kendall to advance the DEAME Framework [24, 49].

## Method

**Fig. 1:**
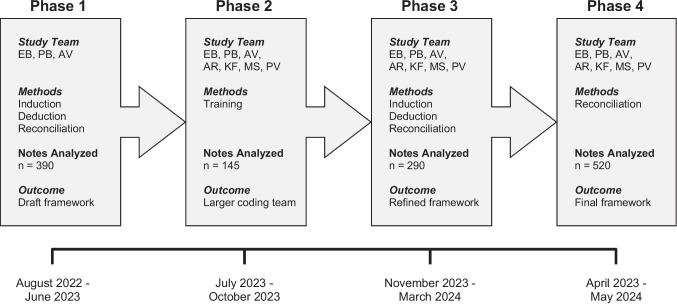
Study overview. Each phase of the four-phase study design lists the study team members involved, the methods used, the number of notes analyzed in the study sample, and the outcome of the study phase

To develop a framework that comprehensively and clearly formalizes the key design features in individual EFs, we assembled a team of experts in qualitative research, education, and medical education. This team incorporated inductive category generation, inductive and deductive code generation, and codebook reconciliation in a four-phase study design (Fig. 1). Our approach was guided by the principles of thematic analysis, which Braun and Clarke define as “searching across a data set to find repeated patterns of meaning”[50]. This effort resulted in a framework built upon a codebook that defines (i) categories that thematically group sets of (ii) codes that detail specific design features of individual EFs.

To develop the framework, we purposively sampled six learner-created Anki decks based on their routine use in UME; five were recommended by learners on Reddit, one is commonly used at our institution, and several have appeared in studies of EF use in UME [51, 52]. The decks vary in structure, size, origin, and targeted learner (see Online Resource 1 for description of study decks). We randomly sampled *notes* from each deck. A *note* is the Anki terminology for the base unit of card creation, and one note may give rise to multiple flashcards (see Online Resource 2 for a schematic of one Anki note creating two flashcards). We analyzed code applications using the Dedoose qualitative coding software and the R programming language [53, 54]. Because one of our categories, *Card Organization*, only differed substantially between decks and did not benefit from continuous cycles of evaluating individual cards, we used only a single round of induction and deduction to develop it as described in Online Resource 3.

Our study was determined exempt from ongoing review by the University of Michigan IRBMED (HUM00222432).

### Phase 1—Initial Framework

Two researchers (EB and PB) engaged in several rounds of open coding on proportionally sampled notes from each of the six study decks, totaling 390. After a completed round of coding, we reconciled code applications and memos, exploring overlap and incongruity in the meaning of categories and codes. After creating a draft codebook with inductively generated categories and inductively and deductively generated codes, we completed an entirely deductive coding round on 100 notes from a single deck (UMMS Preclinical). Discussion after this deductive round was used to align preliminary definitions of the framework’s codes and categories.

### Phase 2—Code Team Expansion

To challenge the codes and categories in the developing framework, we assembled a team of additional coders. Four medical students (MS, AR, KF, PV) with pre-clinical and clinical training and experience with the Anki platform were selected to enrich codebook development. The initial training consisted of two 2-h live sessions that provided definitions, examples, active learning questions, and open discussion of codebook categories and codes. Training sessions were recorded, and training materials were deposited in a shared drive for reference. After initial training, coders participated in five training sets with 5–50 notes to code, totaling 145 notes. Category and code definitions were adjusted throughout the training by incorporating coder feedback from open discussion, deliberation, and memoing. After thorough training and team discussions in phase 2, we proceeded to phase 3 to explore potential gaps in the coding structure and produce a refined framework.

### Phase 3—Refined Framework

Following the successful onboarding of new coders, we split our full coding team into two small groups (group 1: EB, PB, AR, KF; group 2: EB, PB, MS, PV) to encourage active discussion around gaps in the codebook. Each group had regular reconciliation meetings on a set of 30–50 notes, for a total of 290 notes in phase 3. Discussions were guided by coder feedback on coding challenges, intercoder reliability (ICR) calculations suggesting disagreement, and manual inspection of note-level coding disagreements.

We continued coding rounds and discussions until we agreed that we had reached theoretical sufficiency for categories and meaning saturation for codes in the framework [55, 56]^.^ We assessed the theoretical sufficiency of framework categories through small and large group discussions with the study team. We assessed meaning saturation with small and large group discussions, logs of codebook changes, and memoing. When coders agreed that (a) no new categories should be added, and (b) the existing codes were as reasonable as possible given (1) the challenges of sampling such large decks for sometimes rare phenomena and (2) the limitations of studying, e.g., thought processes with this methodology, we completed one final round of coding to assess agreement and proceeded to phase 4.

### Phase 4—Final Framework

All coders read and commented on the final codebook and code examples document. When all comments were addressed, coders certified that they agreed with the contents of the codebook and felt as if it reflected the research team’s work. Training materials, memos, category/code change logs, and meeting notes were reviewed to assess that their content was reflected in the final code dictionary. Though the coding team agreed that saturation had been reached, due to the large size of these decks, two coders (EB and AR) applied the final codebook to a further sample of 520 notes and reached a consensus on every card in the sample.

## Results

Due to the recency of EFs’ rise among medical learners, many medical educators may lack a robust, confident understanding of EFs. Therefore, the primary goal of this manuscript is to present the DEAME Framework to medical educators to empower their involvement in the study and design of EFs. The DEAME Framework was developed from a detailed, theory-informed, inductive-deductive process that draws on state-of-the-art learning theory by incorporating the *NT*. As such, the framework is substantial and highly granular, containing seven categories and 48 codes, many of which will apply to every EF. Table 1 provides a complete overview of the framework and Online Resource 4 displays an example application of the complete framework to a single example flashcard. In the following section, we present each of the seven categories in the framework with a visual aid containing definitions and examples of codes in that category, as well as commentary on possible student-centered design implications. Further description of the framework may be found in our final codebook (Online Resource 5) and examples document (Online Resource 6). At the end of this section, we discuss challenges with the framework, including limitations in using the *NT* to classify EFs.

**Table 1:** Design Elements of Anki for Medical Education (DEAME) Framework overview

Category	Subcategory	Codes	Source^a^
Note Components *What multimedia elements are on the front and back of a flashcard?*	Question Components	Text Emphasis Image Table Audio	Induction only
	Extra Components	Text Image Table Audio Link to External Resource Extra Component in Answer	
Question Format *What type of question is on the flashcard?*		Basic Basic Cloze Cloze Image Occlusion Cloze Image Occlusion Image Selection Information Only	Deduction of Basic, Cloze, and Image Occlusion from Anki; remainder from induction
Answer Format *Which form must the answer take?*		Forced-Choice Partial Complex Nominal Full Complex Nominal or Other Short Answer List	Deduction of Forced-Choice from the *NT*; remainder from induction
Cueing *How does the question inform the user which class of knowledge their answer should be drawn from?*		Direct Parallel	Induction only
Knowledge Domain *What kind of knowledge is the card testing?*	Information	Details Organizing Ideas	Deduction from the *NT* only
	Mental Procedure	Single Rule Tactic Algorithm	
	Psychomotor Procedure	Simple Combination Procedure	
Cognitive Process *How is the flashcard testing a given piece of knowledge?*	Retrieval	Recognizing Recalling Executing	Deduction from the *NT* only
	Comprehension	Integrating	
	Analysis	Classifying Matching Generalizing Analyzing Errors	
	Knowledge Utilization	Decision Making	
Card Organization *How do flashcard decks store and categorize information?*	Deck/Subdeck Structure OR Metadata Tags	Content Area Curricular Exam or Division Third-Party Resource Difficulty Importance Status Format Provenance Deck/Subdeck Structure	Induction only; used documentation from deck creators in addition to flashcards in study sample

^a^*NT*=*New Taxonomy of Educational Objectives* by Marzano and Kendall [24]

### Framework Description

#### Note Components

The *Note Components* category describes the multimedia elements present on an EF and has two subcategories: *Question Components* and *Extra Components* (Table 2). In practice, *Question Components* are multimedia elements on the “front” of an EF that are necessary or supportive to the learner in answering the question. For example, image-based *Question Formats* described in the next section would require an *Image* in the question to be answerable by a learner. In contrast, *Text Emphasis* is not required by any question types but could help the learner quickly identify the key elements of the question. After the learner reveals the answer on the “back” of the card, *Extra Components* can elaborate on why an answer is correct or connect learners to helpful resources.

**Table 2:** *Note Components* definitions and examples

Subcategory	Codes	Definition	Example card front	Example card back
Question Components^a^	Text Emphasis	Text in the question has formatting like bolding or italics, generally to emphasize keywords	“What **class** of drugs does bisoprolol belong to?”	
	Image Table Audio	A media object (i.e., image, table, or audio) is present in the question	“What **class** of drugs does bisoprolol belong to?” *Audio recording pronouncing bisoprolol*	
Extra Components^b^	Text	Text is present in the answer or extra fields to provide explanation or context for the answer		Answer: “Beta-blockers “ Extra Fields: “Beta-blockers have the suffix -lol”
	Image Table Audio	A media object (i.e., image, table, or audio) is present in the answer or extra fields		Answer: “Beta-blockers” Extra Fields: *Table summarizing the indications and side effects of Beta-blockers*
	Link to External Resource	A link/URL is present in the answer or extra fields		Answer: “Beta-blockers” Extra Fields:* Video link to YouTube video about Beta-blockers*
	Extra Component in Answer	Any of the above *Extra Components* is present in the answer itself, instead of extra fields		Answer: “Beta-blockers (suffix = -lol)”

^a^Question Components are present on the front side of an EF

^b^Extra Components are present on the back side of an EF. One or more extra fields may appear below the answer on the back side of the card

The variety of *Note Components* offers significant potential to support learners. For example, to arrive at a medical diagnosis, clinicians need to gather and integrate many types of information, such as patient history, physical exam findings, and laboratory tests. EFs that ask learners to diagnose an illness could replicate this real-life scenario by containing any or all the following: a text summary of the patient history, a video of key physical exam findings, and tables with laboratory findings. Additionally, many students use pre-made decks and therefore may encounter EFs with new material. Abundant explanation via *Extra Components* may allow them to successfully integrate novel material without seeking additional sources. However, when including *Note Components* on an EF, accessibility is a key consideration. For example, screen readers may be unable to parse content within images and, therefore, may exclude visually impaired learners from fully engaging with image-containing EFs, raising the importance of accessible alternatives.

#### Question Format

The *Question Format* category jointly describes the kind of question on an EF (“question type”) and the syntax through which the Anki platform encodes that question (“question encoding”) (Table 3). A key observation is that every type of question can be encoded with at least two different syntaxes. The choice of encoding impacts what the learner sees on the front and back of the card, as well as the process of card creation. For example, the *Basic* format is similar to a traditional paper flashcard in which only the answer, not the question and answer, appears on the back—and must additionally be created by inputting the question and answer into separate text boxes called fields. Conversely, the *Basic Cloze* format uses a single field for card creation and the special syntax “{{c[cloze #]::answer shown on card back in bold typeface::optional answer hint shown on card front in bold typeface}}” such that the question and answer are both visible on the card back (see Online Resource 2 for an example flashcard which uses the cloze syntax). In this way, the *Basic Cloze* format may streamline card review—such that learners do not need to switch back and forth between the question/front and answer/back to review the card fully—and card creation—which may be done in a single field.

**Table 3:** *Question Format* question types, encodings, and examples

Codes	Question type	Question encoding	Example card front	Example card back
Basic	Any except fill-in-the-blank	Question on card front and answer on card back	“What cell type is in the image below?” *Image of a red blood cell*	“Red blood cell”
Basic Cloze	Any except fill-in-the-blank	Cloze^d^	“What cell type is in the image below? **[…]**” *Image of a red blood cell*	“What cell type is in the image below? **Red blood cell**” *Image of a red blood cell*
Cloze	Fill-in-the-blank^a^	Cloze	“There is a **[*…*]** in the image below.” *Image of a red blood cell*	“There is a **red blood cell** in the image below.” *Image of a red blood cell*
Image Occlusion Cloze	Fill-in-the-blank	Image mask(s)^e^	*An image contains a red blood cell and text reading “Red blood cell: average lifespan of 28 days.” The first three words, “Red blood cell” are obscured by a colored rectangle*	*An image contains a red blood cell and text reading “Red blood cell: average lifespan of 28 days.”*
Image Occlusion	Identification^b^	Image mask(s)	*An image of a red blood cell has an arrow pointing from an image label which reads “red blood cell” to the red blood cell. The image label is obscured by a colored rectangle*	*An image of a red blood cell has an arrow pointing from an image label which reads “red blood cell” to the red blood cell*
Image Selection	Identification	Image outline(s)^f^	*An image of several types of blood cells has a green circle drawn around a red blood cell*	*An image of several types of blood cells has a green circle drawn around a red blood cell* “Red blood cell”
Information Only	Information only^c^	Any	“Red blood cell: Average lifespan 28 days, lack nucleus, use hemoglobin to transport oxygen and carbon dioxide.”	Same as card front

^a^Fill-in-the-blank = question asks the learner to impute missing text

^b^Identification = question asks the learner to identify all or part of an image

^c^Information only = no question is asked

^d^Cloze = special syntax “{{c[cloze #]::answer shown on card back in bold typeface::optional answer hint shown on card front in bold typeface}}” which appears to learners as “**[…]**” or “**[optional answer hint]**” on the front of the card and “**answer**” on the back

^e^Image mask = a colored rectangle that covers part of an image, typically a label of a structure

^f^Image outline = a colored outline overlaid on part of an image, typically a structure

The construction of this category as a cross-section between question type and encoding hints at a key observation about EF design: form influences function and vice versa. For example, an EF deck that aims to support learners’ identification of anatomical structures could heavily incorporate *Image Occlusion* and *Image Selection* cards. Therefore, this category can complement the *NT*-derived categories below in guiding the alignment of EF design with curricular goals. Finally, the *Information Only* code in *Question Type* suggests that EFs may be used for information storage and passive learning instead of active learning alone; other design features like *Card Organization* described below may be explored to support learners in this particular use of EFs.

#### Answer Format

The *Answer Format* category describes an answer’s overall length and grammatical complexity (Table 4). Of these formats, *Forced-Choice* is unique in that it does not require the learner to form the answer in their own words but merely choose between already provided answers. The other *Answer Formats*, however, require the learner to craft their answers with varying levels of length and complexity (ranging from short, simple *Partial Complex Nominals* to longer, more complex *Short Answers* and *Lists*). In this way, different *Answer Formats* can likely be used to moderate the average difficulty and time required to answer a question. This observation is supported by the fact that *Forced-Choice* answers, by definition, map to the lowest level *Cognitive Process* described by the *NT*, as discussed further below, while higher-level *Cognitive Processes* often use *Short Answers*. *Answer Formats*, alongside *Question Formats*, therefore complement the *NT*-derived categories below to align EF design with curricular goals. Together, these two categories can also represent known types of assessment questions. For example, a text-based free response question would have a *Basic* or *Basic Cloze* type of *Question Format* and a *Short Answer* type of *Answer Format*. By considering the *Question Format* and *Answer Format* in tandem, card creators can incorporate existing literature on known types of assessment questions and additionally gain insight into rendering them in the Anki platform.

**Table 4:** *Answer Format* definitions and examples

Codes	Definition	Example card front	Example card back
Forced-Choice	Answer is chosen from fixed set of options, either directly enumerated (e.g., multiple-choice) or implied by the question (e.g., yes/no)	“Which of the following describes paralysis caused by damage to the brachial plexus? - Erb’s palsy - Amyotrophic lateral sclerosis - Myasthenia gravis”	“Erb’s palsy”
Partial Complex Nominal^a^	Answer is a subset of a complex nominal	“**[…]** palsy is paralysis caused by damage to the brachial plexus”	“**Erb’s** palsy is paralysis caused by damage to the brachial plexus”
Full Complex Nominal^a^ or Other	Answer is a complete complex nominal or other short phrase like a single noun, adjective, or adverb	“**[…]** is paralysis caused by damage to the brachial plexus”	“**Erb’s palsy** is paralysis caused by damage to the brachial plexus”
Short Answer	Answer contains long phrases or sentences with complex grammar, typically, at minimum, a noun or noun phrase and verb	“What is Erb’s palsy?”	“Paralysis caused by damage to the brachial plexus”
List	Answer contains multiple of any of the other *Answer Types* and typically has a list-like structure indicated by numbers/letters, punctuation, or spacing	“What is the differential diagnosis of Erb’s palsy? **[Top 3]**”	“What is the differential diagnosis of Erb’s palsy? **1) clavicular fracture** **2) osteomyelitis** **3) septic arthritis**”

^a^Complex nominal = a phrase with a noun and a collection of adjectives or nouns representing a single existing entity which we expect a general medical audience to recognize [63]

#### Cueing

The category of *Cueing* describes how or whether the question communicates to the learner from what class of knowledge the answer should be drawn (Table 5). When questions are syntactically interrogative or imperative, they typically necessitate the inclusion of a *Cue* due to their grammatical construction. For example, “Which medication commonly causes chronic cough?” or “Name a medication that commonly causes chronic cough” both suggest that the answer should be drawn from the class of knowledge “medications.” However, when the front of a card instead contains a declarative statement with a fill-in-the-blank/*Cloze* question, a *Cue* may not be present. For example, the *Cloze* question “{{c1::ACE inhibitors}} commonly cause chronic cough” does not contain a *Cue* for the answer “ACE inhibitors,” which are a type of medication. When no or limited assistance is given, we say a question has no *Cueing*. When more robust assistance is given, we describe two types of *Cueing*: *Direct* and *Parallel*.

**Table 5:** *Cueing* definitions and examples

Codes^a^	Definition	Cue specificity	Example card front	Example card back
Direct Cueing	Question contains a word or phrase that specifies the class of knowledge the answer should be drawn from; cues may range in specificity from directly providing the answer choices to select from to including the general class of knowledge	Very specific ↓ Moderately specific	What is the first line ***SSRI*** for bulimia? **[*fluoxetine or*** ***sertraline*]**	What is the first line ***SSRI*** for bulimia? **fluoxetine**
			OR	OR
			What is the first line ***SSRI*** for bulimia?	**fluoxetine**
			OR	OR
			The first-line ***treatment*** for bulimia is **[…]**	The first-line ***treatment*** for bulimia is **fluoxetine**
No Cueing	Question does not contain a word or phrase with sufficient specificity to communicate to the learner which class of knowledge the answer should be drawn from	Minimally specific ↓ Least specific	“Name three ***things*** you ***do*** for patients with bulimia: **[…]**”	Name three ***things*** you ***do*** for patients with bulimia: **CBT, nutritional counseling, antidepressants (esp. fluoxetine)**
			OR	OR
			bulimia = **[…]**	bulimia = **CBT, nutritional counseling, antidepressants (esp. fluoxetine)**
Parallel Cueing	Question contains a word or phrase that belongs to the same class of knowledge that the answer should be drawn from; learners can impute the class of knowledge from this word or phrase	Specificity does not apply	Fluoxetine is first-line therapy for **[…]** and ***OCD***	Fluoxetine is first-line therapy for **bulimia** and ***OCD***
			OR	OR
			Fluoxetine causes ***restlessness***, ***rashes***, and **[…]**	Fluoxetine causes ***restlessness***, ***rashes***, and **joint pain**

^a^*Direct Cueing* and no *Cueing* are situated at opposite ends of a continuum of specificity of *Cue*, with examples of *Cues* (bolded and italicized) provided. *Parallel Cueing* does not exist on this same spectrum and instead relies on words related to the answer to provide the user with a *Cue*. One may note that each example may have multiple *Cues* with similar or different types. For example, the first example of *Parallel Cueing* has a *Direct Cue* (“therapy”) as well as a *Parallel Cue* (“OCD”)

In practice, *Cueing* represents the degree and type of assistance the question provides the learner in knowing what kind of answer the question requires. When questions lack this assistance, they can be challenging (which may be a useful tool for testing a more expert audience), frustrating (because the learner must spend time figuring out what the question is asking, instead of what the answer is), or useless (because the learner cannot figure out what the question is asking, and must reveal the answer to figure it out). Questions with stronger *Cueing*, especially *Parallel Cueing*, may help trigger the answer by encouraging “cued recall,” in which encountering related terms helps learners remember missing information [57].

#### Knowledge Domain

The category of *Knowledge Domain* describes the type of knowledge encapsulated in the question and answer (Table 6). This category is derived from the *NT* and has three subcategories: *Information*, *Mental Procedure*, and *Psychomotor Procedure*. This distinction between informational and procedural knowledge is present not only in the *NT*, but also in curricular design and assessment for medical education [58]. While procedural knowledge might seem challenging to encode in an EF, the *NT* recognizes an overlap between how brains store and engage with *Information* and *Psychomotor* or *Mental Procedure Knowledge Domains*; when a learner first encounters *Procedural* knowledge, they will often encode it in words, similar to *Information*. As they become more expert, they may engage with this knowledge automatically. Therefore, to distinguish *Procedure* from *Information*, the coding team was instructed to use their medical expertise to infer from textual descriptions when a “doer” was present in the question or answer. For example, we coded “What is the definitive diagnostic test for pulmonary embolism?” as a *Mental Procedural* question because it implies that an unnamed clinician must perform a cognitive action to choose the correct diagnostic test, “Pulmonary angiogram.”

**Table 6:** *Knowledge Domain* definitions and examples

Subcategory^a^	Codes	Definition	Example card front	Example card back
Information	Details	Definitions and characteristics of specific persons, places, living and nonliving things, and events	“Gout is characterized by **[…] crystals** in the joint fluid.”	“Gout is characterized by **monosodium urate crystals** in the joint fluid.”
	Organizing Ideas	General statements for which examples could be provided about classes/categories/abstractions of persons, places, living and nonliving things, and events; includes associations and cause-effect relationships	“Men over 65 with **CKD** are likely to develop what rheumatic joint condition?”	“Gout”
Mental Procedure	Single Rule	A single cognitive action OR the circumstance in which that single cognitive action should be performed; an action requires a “doer” of that action, through the “doer” may not be explicitly stated	“What **definitive diagnostic test** should be ordered for suspected gout?”	“Joint fluid analysis”
	Tactic	Multiple *Single Rules* which combine in no prescribed order to form a complete process for completing a cognitive task OR the circumstance in which those rules should be used	“What **panel** of diagnostic tests should be ordered for suspected gout?”	“- Joint fluid analysis - Blood/urine uric acid - ESR/CRP - Joint imaging”
	Algorithm	Multiple *Single Rules* which combine in a prescribed order to form a complete process for completing a cognitive task OR the circumstance in which those rules should be used	“What is the **treatment algorithm** for gout?”	“If no NSAID risk factors -> NSAIDS, otherwise: Monoarticular + injectible joint -> intra-articular corticosteroids Polyarticular or non-injectible joint -> oral steroids/colchicine”
Psychomotor Procedure	Simple Combination Procedure	The steps to perform a simple task comprised of physical motions OR the circumstance in which that task should be performed	“What are the **steps** to perform the Babinski maneuver?”	“- Seat/recline patient - Hold heel in one hand - Firmly stroke bottom of the foot from heel to big toe with bottom of reflex hammer”

^a^Subcategories and codes are derived from the *New Taxonomy of Educational Objectives* by Marzano and Kendall [24]

Medical learners must engage with many different types of knowledge during their studies. By recognizing the presence of *Information*, *Mental Procedures*, and *Psychomotor Procedures* in EFs, educators may better be able to target specific learning objectives and select complementary design elements to encourage their mastery.

#### Cognitive Process

The *Cognitive Process* category, also adapted from the *NT*, describes the type of thinking a learner might engage in to generate the answer (Table 7). In other words, how does a question test knowledge? *Retrieval* is the least complex subcategory requiring the learner to generate an answer but not demonstrate an “in-depth” understanding of the “basic structure…or critical and noncritical elements” of the knowledge [24]. The next most complex *Cognitive Process*, *Comprehension*, requires the learner to demonstrate this “in-depth” understanding of the concepts underlying the answer, such as how concepts of *Information* are related or why a *Procedure* works. The final two *Cognitive Process* subcategories require using this “in-depth” knowledge to reason and generate new knowledge. The subcategory *Analysis* requires the learner to reason to “generate new conclusions,” and the subcategory *Knowledge Utilization* requires the learner to reason to “apply…knowledge in specific situations.” Of the 13 codes shared between these four subcategories, we include nine in the DEAME Framework. Of the four excluded codes, we constructed draft definitions in our codebook for three that we felt could reasonably be rendered in the EF format but did not encounter in our sample (Online Resource 7).

**Table 7:** *Cognitive Process* definitions and examples

Subcategory^a^	Codes	Definition	Example card front	Example card back
Retrieval	Recognizing	The user selects the answer from a set of provided or implied answer choices (i.e., a *Forced-Choice* answer)	“Is gout characterized by **monosodium urate crystals** in the joint fluid?”	“Yes”
	Recalling	The user produces an answer from memory, but does not necessarily understand how or why that answer is correct	“Gout is characterized by **[…] crystals** in the joint fluid.”	“Gout is characterized by **monosodium urate crystals** in the joint fluid.”
	Executing	The user performs a procedure without significant error but does not necessarily understand how or why the procedure works	“Compute the **corrected calcium** for the CBC results below.” *Table with CBC results*	“12.2 mcg/ml”
Comprehension	Integrating	The user identifies how or why two pieces of knowledge are connected	“What is the **pathophysiology** of gout?”	“Elevated uric acid -> monosodium urate crystal deposition in joints -> joint pain/inflammation”
Analysis	Classifying	The user identifies superordinate and/or subordinate categories related to multiple pieces of knowledge	“Rheumatoid arthritis, systemic lupus erythematosis, and gout all belong to which **group** of diseases?”	“Inflammatory arthropathies”
	Matching	The user identifies important similarities and differences between multiple pieces of knowledge	“How do you **distinguish** between gout and pseudogout?”	“Gout: monosodium urate crystals, small joint distribution, elevated uric acid levels Pseudogout: calcium pyrophosphate crystals, large joint distribution, normal uric acid levels”
	Generalizing	The user constructs new “rules-of-thumb” or associative/causal relationships from existing knowledge	“What can the pathophysiology of gout and rheumatoid arthritis likely tell you about how all inflammatory arthropathies develop?”	“Damage to joint epithelial cells causes release of pro-inflammatory mediators like cytokines which can create a positive-feedback cycle of further joint destruction and inflammation”
	Analyzing Errors	The user identifies whether something is true/correct or false/incorrect and explains why	“Pseudogout is characterized by elevated uric acid levels. T/F and why?”	“False. Gout is characterized by elevated uric acid levels. Pseudogout may have elevated calcium levels, but has normal uric acid levels”
Knowledge Utilization	Decision Making	The user uses existing knowledge to make a decision in a specific context	“A 65yo man with diabetes and CKD presents with acute swelling, pain, and warmth in his right DIP joint. What is the likely **diagnosis**?”	“Gout”

^a^Subcategories and codes are derived from the *New Taxonomy of Educational Objectives* by Marzano and Kendall [24]

Several important observations emerged while applying the *NT Cognitive Processes* to EFs. First, different students may employ different *Cognitive Processes* on the same card depending on their prior experience with the material. For example, if a student already knows that Myasthenia Gravis and Lambert-Eaton Syndrome belong to the category Neuromuscular Junction Diseases, then they may simply *Recall* that relationship instead of newly creating it in their minds with the *Classifying Cognitive Process*. When multiple *Cognitive Processes* were possible for a given card—typically a *Retrieval* and a higher-level *Process*—we selected the most complex one. A related observation is that while these *Cognitive Processes* are ordered by complexity, that does not mean they are ordered by difficulty. For example, using a *Retrieval* process in a multiple-choice question about advanced clinical material with which a learner is unfamiliar may be easier than using a more complex *Cognitive Process* on more foundational information she learned recently [24]. Nonetheless, the two *NT*-derived categories of the framework provide exciting avenues for student-centered and curriculum-aligned EF design. For one, the presence of cards which may elicit higher-order thinking like *Decision Making*, suggests that EFs may be able to support more advanced medical learners in graduate medical education (GME) or continuing medical education (CME).

#### Card Organization

The *Card Organization* category describes the two hierarchical labeling systems that can classify, arrange, or order EFs (Table 8). In Anki, each EF is assigned on creation to a location in an EF deck or subdeck (i.e., *Deck/Subdeck Structure*) and may optionally be assigned *Metadata Tags*. Neither organizational system appears by default to the learner on either the front or back of individual flashcards but may be used to select specific cards for review. Indeed, this function may be very valuable to support learners given the expansive size of some popular EF decks (in our study, ranging from approximately 15,000–31,000 cards as shown in Online Resource 1). Different populations of learners may benefit from using *Card Organization* labels to review decks in specific and measured ways, including selecting cards relevant to their current curriculum (e.g., through *Content Area* and *Curricular Exam or Division* labels) or prioritizing important cards for emergent review (e.g., through *Importance* labels). Additionally, *Card Organization* labels that serve more clerical functions (e.g., *Status* and *Provenance*) could help support the ongoing improvement of EFs deck, particularly through crowd-sourcing models.

**Table 8:** *Card Organization* definitions and examples

Subcategory	Codes	Definition	Example
*Deck/Subdeck Structure* OR *Metadata Tags*	Content Area	Labels^a^ that suggest content is related to general (e.g., medical specialties) or specific (e.g., classes of drugs) content areas	Rheumatology > Inflammatory Arthropathies > Gout
	Curricular Exam or Division	Labels that suggest content is related to specific exams (e.g., medical boards) or curricular divisions (e.g., pre-clinical sequences/courses)	Step 2 > Internal Medicine Shelf
	Third-Party Resource	Labels that suggest content is related to third-party resources, including specific pages/videos/aspects of those resources	Sketchy Pharmacology > Anti-inflammatory Drugs > Gout Drugs
	Difficulty	Labels that suggest content has a certain anticipated difficulty or rudimentariness	Foundational
	Importance	Labels that suggest content has more or less value or importance	High-Yield
	Status	Labels that suggest content should be managed in a certain way, (e.g., be deleted)	Duplicated > Delete
	Format	Labels that suggest content is rendered in a particular format (esp. a proprietary format)	Image Selection
	Provenance	Labels that suggest which user or group of users created or edited a card	Zanki
	Deck/Subdeck Structure (*Metadata Tags* Only)	*Metadata Tags* which duplicate all or part of the *Deck/Subdeck Structure*	AnKing > Zanki

^a^A label may appear in a hierarchical tree that describes the location of an EF (*Deck/Subdeck Structure*) or connect it to cards with similar labels across decks/subdecks (*Metadata Tags*). Any one label may serve more than one purpose, such as the label “Low-yield Surgery” suggesting a *Content Area* (“Surgery”), *Curricular Exam or Division* (“Surgery,” e.g., a Shelf exam), and *Importance* (“Low-yield”)

### Framework Challenges

All frameworks represent an artificial way to apply organized representations to complex real-world phenomena. Frameworks are, by definition, never complete and may need consistent refining to continue to achieve their stated goals. In this way, the framework presented in this manuscript was designed in such a way as to allow for its future modification and extension. This section outlines the challenges we faced in developing the framework.

For elements of the DEAME Framework that were derived from the *NT*, some of the subcategories and codes in the *NT* did not apply to the EF formats observed. For example, the only *Psychomotor Procedure* code that the coding team agreed could appear on a flashcard is a *Simple Combination Procedure*, which describes physical motions performed together to complete a simple task; other codes in the *NT* for Psychomotor Procedure felt either too embodied (e.g., a *Complex Combination Procedure*, like playing basketball) or too basic (e.g., *Foundational Procedure*, like body posture or balance) to be reasonably assessed through the flashcard medium. Additionally, the *NT* contains two high-level *Cognitive Processes*–*Self-system Thinking* and *Metacognition*–describing the relationship between knowledge and a learner’s motivations, emotions, and beliefs. While EF platforms themselves may support engagement with these *Cognitive Processes*, the coding team concluded that individual questions and answers on EFs were unlikely to support these ways of thinking.

Additionally, some elements of the framework appeared to apply to EFs but offered barriers to consensus building among the coding team. The *Cueing* category, for example, presented challenges in creating a clear boundary between *Cues* specific enough to be *Direct Cues* and *Cues* that offered no *Cueing*. Indeed, this boundary may change depending on the expertise of the learner. For example, a cardiologist may not have trouble selecting a limited number of likely candidate answer classes for “What do you do for heart failure?”, but a medical student might. We encountered similar challenges creating boundaries between the *NT*’s types of *Information*, specifically of *Facts* (a kind of *Detail*) and *Generalizations*/*Principles* (two kinds of *Organizing Ideas*), as they applied to EFs. A key challenge here was the tension between the semantics of the question and our team’s understanding of the content. For example, imagine a card that tests symptoms of upper respiratory infections (URIs). This card could be worded as “Rhinorrhea is a symptom of {{c1::URIs}} in > 80% of cases,” which is a *Fact*; this statement illustrates a specific characteristic of a person, place, or thing (in this case, a symptom, rhinorrhea). However, worded another way, “Most (> 80%) {{c1::URIs}} cause rhinorrhea” reads as a *Principle*, which is a specific type of *Generalization* that describes cause and effect relationships. This tension between the semantics of how content is represented and the content itself is difficult to resolve in flashcards, which often contain short questions, and between coders who may have a different subjective understanding of the content. Despite this lack of overall agreement, subsets of coders could consistently apply these codes with moderate or high agreement.

In both examples, *Direct* vs. no *Cueing* and *Details* vs. *Organizing Ideas*, the coding team felt as if there were some palpable differences in how each could impact an EF learner, though we could not reach an absolute agreement. Therefore, we have retained the codes in the framework and encourage future work to improve their definitions using methods suited to this task.

## Discussion

Medical learners have increasingly relied on EFs as a study tool in recent years. Preliminary research suggests that learners may engage with and benefit from EFs differently than traditional paper flashcards. Therefore, it is critical to explore the current state of EFs in medical education and how they can be designed to support curricula and learning processes. Such explorations have hitherto been limited by a lack of understanding of EF’s educational content and design. Therefore, in this study, we introduce the Design Elements for Anki in Medical Education (DEAME) Framework, which offers seven thematic categories that propose a foundational terminology to describe what design elements EFs may contain.

The DEAME Framework supports the premise that past understandings about paper flashcards may not apply wholesale to EFs. For example, unlike paper flashcards, EFs can integrate videos into questions, answers, and explanatory information to support advanced audiovisual learning. Additionally, EFs allow knowledge to be highly connected to other EFs using metadata or to general medical knowledge bases using electronic links. Finally, we observed EFs in our sample which could be categorized under as procedural knowledge and higher-order cognitive processing according to the *NT*. This supports preliminary evidence that EFs may not simply offer rehearsal of factual knowledge. Besides these, many other categories in the DEAME Framework may shed light on how students might benefit from EFs currently in everyday use.

Beyond offering insights into the current state of EF design, the DEAME Framework may also provide educators, researchers, and learners a launch pad to improve the value of EFs as a learning tool.

The DEAME Framework may offer learners greater awareness of EF design and how it might influence their learning. For example, more advanced learners may use DEAME to identify card designs that could elicit more complex cognitive skills like decision-making. This aligns with previous work that suggests that fostering student *metacognition*, including teaching learners about the study tools they use, may improve their academic performance [59–61]. However, while we may empower students to control their own individual learning, we need involvement from other stakeholders with a background in pedagogy to fully realize the potential of EFs as a learning tool. Namely, these stakeholders should focus efforts on remedying common learner concerns around EFs: low-quality cards that poorly align with curricula [4, 8, 28].

The DEAME Framework may empower education researchers to define principles and best practices of high-quality EF design. For example, which *Question Types* might be most effective in teaching identification of anatomical structures vs. application of those anatomical structures to clinical cases? Because EF platforms like Anki generally contain granular usage metrics, empirical studies that compare design choices on learner outcomes could be performed quickly [52]. Notably, existing studies of EFs largely exclude descriptions of card designs and offer limited insight into effective EF design. The DEAME Framework provides a missing terminology to communicate EF design choices and may enhance the interpretability and replicability of future EF studies.

For educators, the DEAME Framework represents a new connection forged between EFs and learning theory that motivates future work to develop curriculum-aligned EFs. A handful of prior studies have used expert review to align EFs with specific curricular or learning objectives, but none provide explicit guidance on performing this alignment [22, 43, 62]. The DEAME Framework supports the development of a formal process for such alignment because it connects the *NT*, a framework for understanding educational objectives, to other elements of EF design. For example, an EF with a *Recognizing Cognitive Process* must necessarily have a *Forced-choice Answer Type*. Similar relationships between different parts of the DEAME Framework could be further explored with quantitative or other qualitative methodologies to inform EF alignment with curricular content.

In practical terms, institutions can leverage DEAME Framework—and future insights into high-quality, curriculum-aligned EF design that it motivates—as a starting point for robustly implementing EFs as part of their educational offerings. For example, a faculty member or instructional designer could leverage the framework to audit student-created EF decks and supplement them with additional cards to ensure comprehensive coverage of learning objectives. Additionally, institutions could use the DEAME Framework to facilitate faculty development around EFs so that faculty may provide specific and evidence-based guidance to students using EFs. Furthermore, elements of the DEAME Framework could be considered when drafting institutional policies around parallel curriculum and eLearning resources, such as reuse of third-party multimedia in EFs. When engaging in any of these example initiatives, we encourage educational leaders to consolidate their efforts across institutions to ensure equitable and efficient resource distribution. For example, platforms like AnkiHub exist to enable collaborative design and sharing of EFs among learners and educators everywhere [27].

A key strength of the DEAME Framework is that it connects EFs to a rich tradition of educational thought and research by its extension of the *NT*. Notably, this extension is a novel one, in that it represents the first time active learning questions contained in EFs have been aligned to the *NT*’s domains. Overall, we found this application of the NT to EFs for UME to be reasonably simple and highly powerful. For example, we feel the granularity of the *Cognitive Processes* in the *NT*, compared to other taxonomies like Bloom’s, appropriately captures the breadth of questions we encountered in our decks. Our application of the *NT* to EFs was eased by the *NT’s* rich description and abundant examples, particularly because these were done in using the cross-sectional relationships between *Knowledge Domains* and *Learning Processes* [49].

Our study has several limitations that motivate continued development. While we aimed to sample various decks widely used by the UME population, we may have omitted decks with important or unique design elements. Similarly, the scope of our project excluded aspects of EFs, such as the spaced repetition algorithms integrated in EF platforms, which may meaningfully impact learner outcomes and which educators may wish to consider when designing educational interventions with EFs. Additionally, more specific methodologies may be needed to explore the relationship of the *NT*’s domains to EFs, as learners may contextualize and engage with knowledge differently between individuals and across time. Such exploration could also clarify which medical skills and knowledge are best taught and reinforced by the EF medium. Notably, this framework can be easily modified or extended to encompass new understandings about EF design in medical education and beyond.

## Conclusion

Use of EFs among medical learners is substantial and continues to rise every year. Given that medical students who use EFs typically use them for hundreds to thousands of hours across their years of training, EFs represent a promising opportunity to improve the capacity of our future physician workforce to learn in a rapidly evolving knowledge ecosystem. The DEAME Framework offers a critical foundation to encourage the involvement of expert educators, researchers, and clinicians in developing and evaluating EFs for the next generation of medical learners. In presenting the DEAME Framework, we have asserted a foundation to guide future research into the design implications of EFs, provided language for the reproducibility and comparison of studies of EFs on educational outcomes, and offered a way for learners and educators to align EFs with curricular learning objectives.

## Data Availability

The data that support the findings of this study are available from the corresponding author upon reasonable request.
